# Notes on Chloral

**Published:** 1870-12

**Authors:** H. Y. Evans


### Selections.

Notes on Chloral.—By Dr. H. Y. Evans.—It seems to me that the remark so often made "that we are governed by fashion in medicine" is an erroneous one. The underlying cause of the disposition to change and drop certain remedies after a period of varying success is owing, in a great degree, to the use of an inferior preparation. The numerous reported failures of the use of hydrate of chloral seem to make this fact especially true in regard to this drug.

I have noted the effects of this drug in twenty-four consecutive cases.

In the first *sixteen* cases (in doses of grs. xxv to xxx) its effects were really delightful.

In the *seventeenth* and *eighteenth* it failed in producing anything but delirium and a subsequent headache.

In the *nineteenth*, *twentieth*, and *twenty first* cases the effects were entirely satisfactory.

In the *twenty-second* (nephralgia), grs. xxx, repeated every hour for three hours, resulted in wakefulness and headache.

The use of it in the *twenty third* and *twenty-fourth* cases resulted, within an hour, in vomiting and delirium, and, at the expiration of eight hours, a heavy, unpleasant sleep.

Cases *seventeen*, *eighteen*, *twenty-three*, and *twenty-four* were most suitable cnes for happy effects. The failure, therefore, made me anxious to discover the cause. On inquiry as to the character of the preparations used in these cases, I was convinced that three out of four of my failures were directly due to the use of an inferior and deleterious drug. These preparations had a heavy, dead, camphory odor, and, in one instance, a dirty appearance.

The fact mentioned by Dr. Baldwin—that a small dose (grs. xv) largely diluted (in f℥ij of fluid) seems to act *more promptly* and more pleasantly than a large (grs. xxx.) one sparingly diluted (in fʒiij of fluid)—is an important one; and I am so convinced of its truth that I invariably act upon it.—*Medical Times.*

